# Leafcutter ants adjust foraging behaviours when exposed to noise disturbance

**DOI:** 10.1371/journal.pone.0269517

**Published:** 2022-06-08

**Authors:** Briony Byrne, Selvino R. de Kort, Scott M. Pedley

**Affiliations:** Ecology and Environment Research Centre, Department of Natural Sciences, Manchester Metropolitan University, Manchester, United Kingdom; University of Pavia, ITALY

## Abstract

We investigate the impact of anthropogenic noise on the foraging efficiency of leafcutter ants (*Acromyrmex octospinosus*) in a controlled laboratory experiment. Anthropogenic noise is a widespread, pervasive and increasing environmental pollutant and its negative impacts on animal fitness and behaviour have been well documented. Much of this evidence has come from studies concerning vertebrate species with very little evidence for terrestrial invertebrates, especially social living invertebrates. We compare movement speed, forage fragment size, and colony activity levels of ants exposed to intermittent elevated noise and in ambient noise conditions. We use intermittent and temporally unpredictable bursts of white noise produced from a vibration speaker to create the elevated noise profile. Ant movement speed increased under elevated noise conditions when travelling to collect forage material and when returning to the colony nest. The size of individually measured foraged material was significantly reduced under elevated noise conditions. Colony activity, the number of ants moving along the forage route, was not affected by elevated noise and was consistent throughout the foraging events. Increased foraging speed and smaller forage fragments suggests that the ants had to make more foraging trips over an extended period, which is likely to affect energy expenditure and increases exposure to predators. This is likely to have significant fitness impacts for the colony over time.

## 1. Introduction

Anthropogenic noise is a significant environmental pollutant and is increasingly recognised in environment legislation [1, 2]. Noise such as industrial development, motorised transport and energy-extraction infrastructure can vary greatly from natural sounds by typically having lower frequencies, higher amplitudes and occur more frequently [3, 4]. A wide range of negative biodiversity impacts have been documented, including disruption to conspecific communication and physiological processes, decreased reproductive success, and inefficient resource acquisition, all of which may contribute to the decline of wildlife [1, 4].

The overwhelming majority of anthropogenic noise studies have been conducted on avian and mammalian fauna, with very few studies assessing impacts on terrestrial invertebrates, especially social invertebrates [2, 5]. Invertebrates provide vital ecosystem services and are major biotic components in all ecosystems, and therefore assessing impacts of anthropogenic disturbance on invertebrates is important. Additionally, since invertebrates use both airborne and substrate-borne sound, and they have a wide range of sensory structures to detect sound the impact of anthropogenic noise is likely to be unique and different from the model systems studied so far [6]. Furthermore, as much anthropogenic noise overlaps with the frequency responses of invertebrate hearing there is potential for significant impact to the auditory capacity for many invertebrate species [2, 4, 7]. Previous work on invertebrates has demonstrated interference of male courtship signals (substrate striking and palp stridulation) and mating success in wolf spiders (*Schizocosa ocreata*) under elevated noise conditions [8], and changes to arthropod community structure with elevated airborne and substrate-borne noise from gas extraction [9]. Evidence for call modification in noisier environments, similar to many avian and mammal studies [1], has also been reported for grasshoppers [10] and cicada [11], where both taxa increased their call frequencies in response to increased background noise. To date, no data was published on the impact of noise disturbance on foraging behaviour of social living invertebrates.

Leafcutter ants (genera *Atta* and *Acromyrmex*) are social insects that use a central place foraging strategy to attain leaf matter with which to cultivate symbiotic fungi that serve as food for the ant larvae. Leafcutters are a good model organism for studying behavioural changes due to their well-defined foraging behaviours, the ease of maintaining laboratory colonies, but also because of their important role as ecosystems engineers and their considerable overall biomass [12, 13]. Cutting and transporting leaf fragments is an energy expensive task [14, 15] and ants can optimise their foraging strategy by regulating loads to account for changes in the environment [16–18]. The use of pheromones in leafcutter foraging communication is well known, but stridulation appears important in *Atta* colony foraging as well [19–22] but in *Acromyrmex* it has not been described. Although it remains uncertain whether ants can perceive airborne pressure waves, there is no uncertainty about whether they can perceive substrate born waves.

Here we use a controlled laboratory experiment to test if and how elevated intermittent noise disturbance affects the foraging behaviours of leafcutter ants (*Acromyrmex octospinosus*). We use *A*. *octospinosus* as a central place forager that has a relatively narrow forager size range compared to other leafcutter species [23], thus potentially needing greater individual flexibility in foraging behaviour [24]. We compared foraging behaviour during elevated noise treatments of temporally variable intermittent white noise to ambient noise conditions as a control. By assessing ants’ movement speed, colony activity counts and size of foraged leaf fragments we investigate if leafcutter ants alter their foraging behaviour when their acoustic environment is experimentally manipulated by adding artificial noise.

## 2. Methods

### (a) Study system

All individual ants tested belonged to the same colony of leafcutter ants (*A*. *octospinosus*), which were obtained from a commercial supplier in the UK. *A*. *octospinosus* naturally ranges from southern Mexico through central America and into Northern South America. Since July 2017, a stable colony (1500–2000 workers) has been kept in the laboratory with the nest housed at a controlled temperature of 25°C at 80% humidity inside a Froilabo climate chamber (model SP-BVEHF). As in previous studies we used a single captive colony to investigate ant behaviour [15, 16, 24, 25], any differences recorded between individuals may only reflect variation in worker behaviour/personality, and not colony level personality, and we acknowledge that colony personality may provide a range of responses [26, 27]. The main focus of the current study is to investigate possible responses in individual worker foraging behaviour, which will provide a base for studies designed to incorporate colony level personality.

The climate chamber housing the nest was connected by 4m of transparent plastic tubing (2.8cm diameter) to three glass boxes (two 30x20x20cm boxes, and one 29x20x6cm). The three boxes were separated by 50cm of plastic tubing that allowed ants to travel between each box to collect forage material and return to the nest via the same route. The first box, furthest from the nest, was used as the forage box where 35g of privet leaves (*Ligustrum ovalifolium*) was placed at the start of each experiment. Privet was obtained from a single source located away from roads and sources of pesticide. The second box in the system was empty and used to record ants from above, having been prepared with Fluon® to restrict ants to the box floor. Two GoPro Hero 7 cameras were used for recording foraging ants. One camera was placed between the first and second glass box and filmed a 20cm section of tubing which was used to record ant numbers. The second camera was positioned directly above the empty second glass box and was used to record movement speed. The third glass box had a raised gravel floor and removable lid and was used to extract leaf fragments from ants.

### (b) Behavioural recording

All experimental replicates (n = 10 noise, n = 10 controls) were conducted between July-August 2019 with only a single replicate performed in any one day (between 8-10am) and random assignment of treatments per replicate. A vibration speaker (Adin B1BT 10W, 80Hz-18kHz) was placed on top of the second glass box. Vibration speakers transduce the electric energy into mechanical energy and make the surface they are attached to vibrate. The glass surface then transmits sound waves into the environment. We used a vibration speaker as it is unresolved whether ants can perceive airborne soundwaves, but they can perceive substrate waves [20]. During noise treatments the speaker played intermittent white noise with LZF_max_ = 98.1 dB(Z) or LAF_max_ = 80db(A) at 10cm from the glass surface, which corresponded to LZF_max_ = 119 dB(Z) or LAF_max_ = 101 dB(A) inside the box. LZF_max_ is the maximum sound level measured using the fast time weighting response and Z frequency weighting. Z—weighting refers to Zero frequency weighting, which implies no weighting across the sound spectrum. A—weighting as used in LAF_max_ is frequency weighted to conform to a notional human hearing response. The latter is commonly used to report sound levels, but as the frequency response of ant hearing is not known, we also report unweighted sound levels here. Sound level was measured using a Casella CEL-63X sound level meter (Fast response). The playback file consisted of 63 intermittent bursts of white noise (WAV format), all with the same peak amplitude, on a one-minute loop. The sound bursts varied randomly in duration from 0.01–2 seconds to avoid habituation. We do not attempt to recreate a specific anthropogenic noise profile (e.g., traffic noise); this study uses an intermittent white noise, therefore covering a wide range of frequencies, set at a relatively high level compared to ambient conditions to elicit potential behavioural modification. In control treatments the speaker remained in place and turned on, but with no noise playing. Ambient noise during the control trials was recorded at LZF_max_ = 76.6 dB(Z) or LAF_max_ = 41dB(A).

To record ant activity counts, video recording was initiated once the first ant entered the forage box. The video recording was conducted over a two-hour period per replicate; roughly the duration it took for the colony to remove >95% of the forage material in pilot studies. During each two-hour recording period, six episodes of video recording were completed, with each episode lasting ten minutes and occurring every other ten minutes. This resulted in six discrete time points of footage per replicate. Videos were analysed using BORIS software [28] with counts made for ants moving towards the forage without leaf material, and ants moving towards the nest carrying leaf material.

To record straight-line movement speed, footage from the second camera positioned above the second glass box was used. Ants were timed moving between the two entry points at either end of the box (24 cm). Timings were only made for ants that moved continuously. If an ant was stationary for more than five seconds, or made directional change greater than 90^o^, observations were stopped, and data omitted from analysis.

### (c) Leaf measurements

Leaf fragment surface area and dry weight was sampled from ants carrying leaf fragments from the forage box to the nest. Fragment extraction was performed by hand from the third glass box using flat-tip tweezers to hold the leaf and a paintbrush to separate ants. Ten leaves were sampled during each of the six ten-minute periods between video recordings. To obtain fragment surface area, leaves were scanned and individual surface area was calculated using ImageJ [29]. Leaf dry weight was used rather than wet weight to avoid inconsistencies due to leaf desiccation. Leaf fragments (n = 10) were pooled within each of the six time points and dried at 45°C for 48 hours before being weighed.

### (d) Statistical analysis

Due to non-normal data distributions non-parametric Wilcoxon tests were used to compare movement speeds and leaf metrics between control and noise treatments. To test these metrics between time points (1–6) non-parametric Kruskal Wallis tests were used, and where significant differences were found pairwise Wilcoxon test were used to test pairwise differences. T-tests were used to test ant activity counts between each time point. Data analyses were performed using R (v3.6.1) [30].

## 3. Results

**Fig 1:**
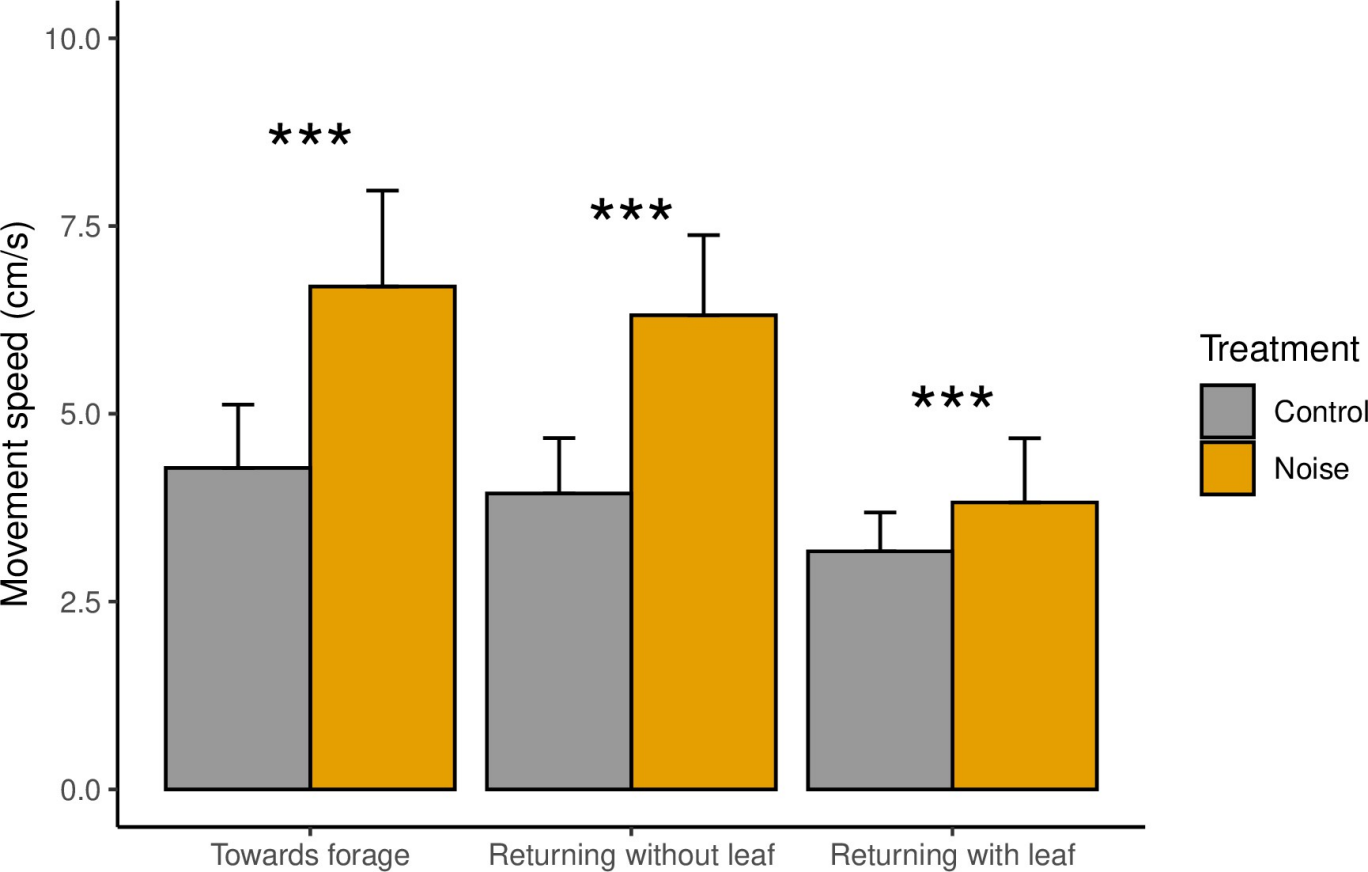
Mean (±SD) straight-line movement speed for three foraging ant behaviours for noise and control treatments. Stars indicate test significance level for pairwise testing between control and noise treatment for each foraging behaviour (*** = P<0.001).

Straight-line movement speed was significantly increased under noise treatments compared to controls (Fig 1, W = 258668, P<0.001), and this was consistent across all the foraging behaviours; towards forage (W = 8018, P<0.001, n = 768), returning to nest without leaves (W = 4400, P<0.001, n = 812), and returning to nest with leaves (W = 43632, P<0.001, n = 745).

**Fig 2:**
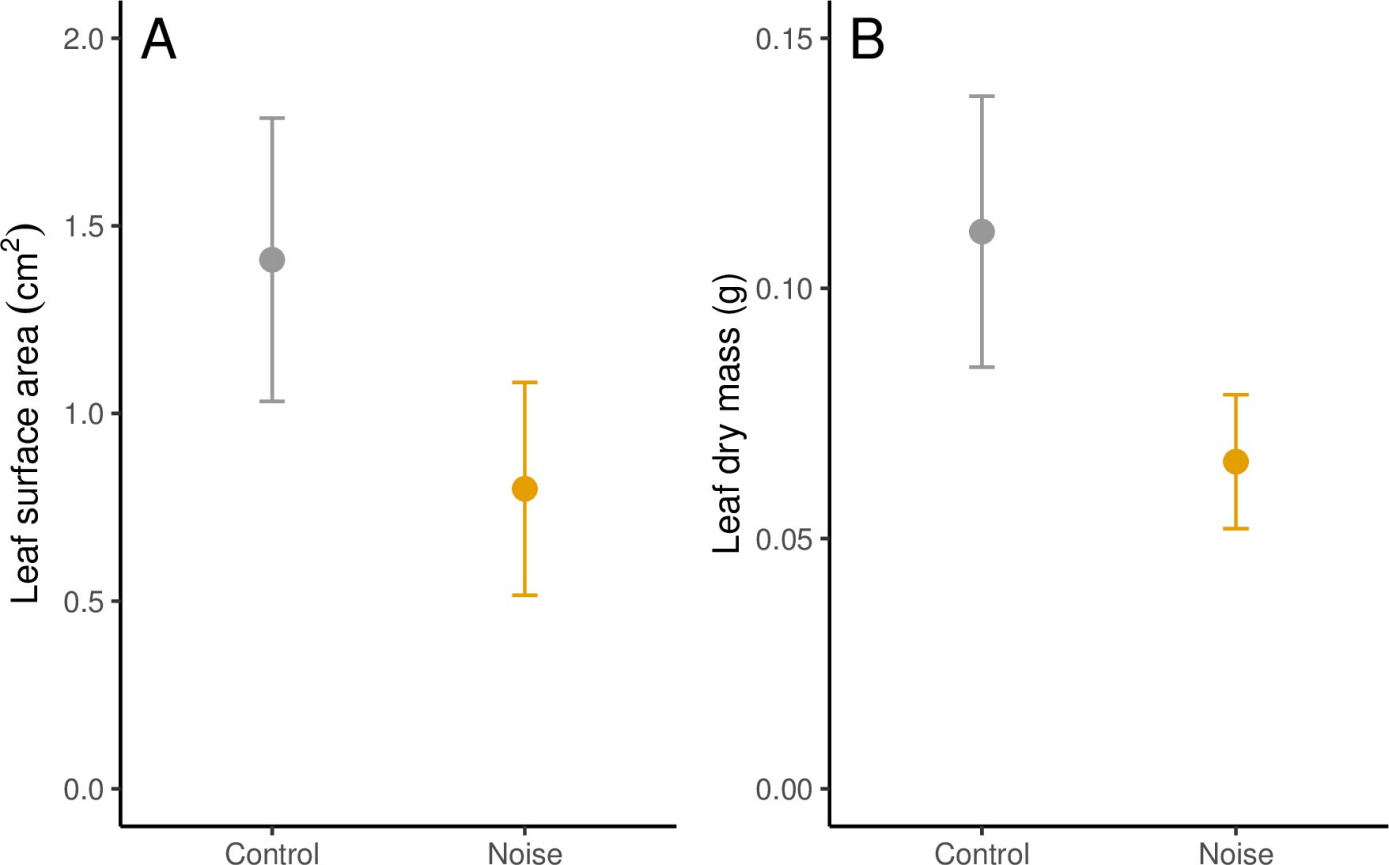
Mean (±SD) of foraged leaf fragment A) surface area (cm^2^) and B) dry mass (g) by *A*. *octospinosus* under noise and control treatments.

**Fig 3:**
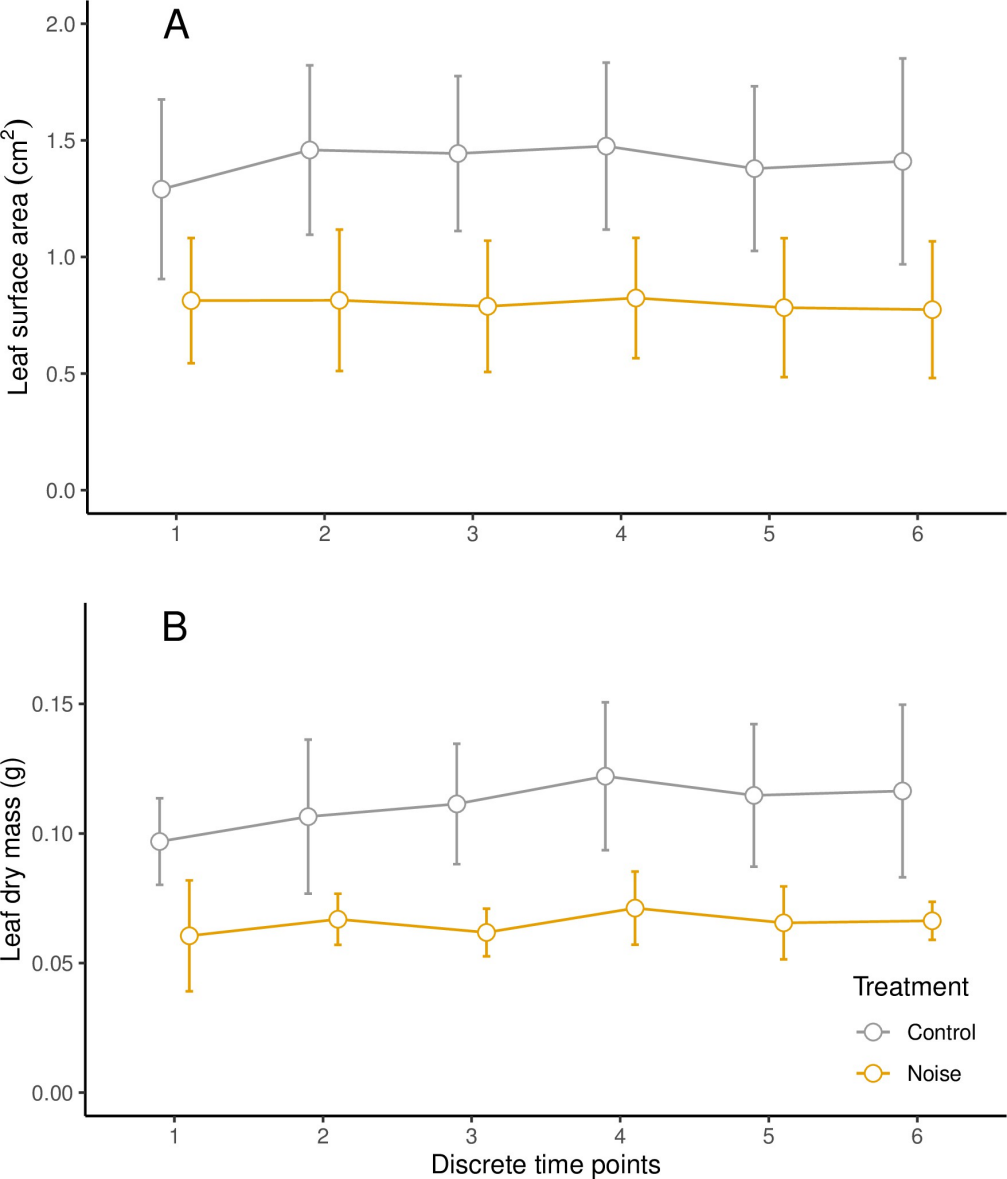
Mean (±SD) of foraged leaf fragment A) surface area (cm^2^) and B) dry mass (g) by *A*. *octospinosus* under noise and control treatments recorded at six discrete time points within each foraging replicate (n = 10).

The surface area (cm^2^) of leaf fragments was significantly smaller in noise treatments compared to controls (W = 324028, P<0.001, Fig 2A). For the noise treatments, fragment surface area was consistent across all recorded time points (Chi = 3.945, P = 0.557). However, fragment surface area for controls was significantly different between time points (Chi = 19.20, P = 0.002) with fragments being significantly smaller in the first time point compared to time points 2–4 (P<0.05, Fig 3A). The dry mass (g) of foraged fragments was also significantly less for noise treatments compared to controls (W = 3520, P<0.001, Fig 2B), but was consistent between time points for both treatments (P>0.05, Fig 3B). S1 Fig in the supporting information shows that leaf metrics (surface area and dry mass) for noise treatments were consistent across all ten replicates, but controls showed much more variation across replicates.

Finally, colony activity, as measured by counts of ants travelling to the forage material and ants returning to the nest with leaf fragments was assessed at each time point. No differences in the numbers of ants travelling to the forage, or ants returning with fragments was found between control and noise treatments (Table 1, P>0.05).

**Table 1:** Mean (±SD) ant activity counts for each recorded time point during control and noise treatments.

Behaviour	Time point (mins)	Control	Noise	t-value	P-value
Towards forage without leaf	0–10	61±27	61±40	0.01	0.995
	20–30	209±108	175±107	0.71	0.486
	40–50	187±85	190±92	-0.06	0.954
	60–70	189±79	184±72	0.14	0.889
	80–90	183±80	174±56	0.29	0.777
	100–110	162±67	173±39	-0.44	0.669
Towards nest with leaf	0–10	4±3	10±15	-0.81^+^	0.434
	20–30	85±50	72±47	0.71^+^	0.485
	40–50	121±40	111±46	0.53	0.600
	60–70	113±23	102±36	0.83	0.422
	80–90	96±24	100±29	-0.33	0.745
	100–110	88±21	87±24	0.02	0.985

Test statistics are given for the comparisons between control and noise treatments. ^+^ indicates square root transformation.

## 4. Discussion

Ants exposed to intermittent and temporally variable white noise foraged smaller leaf fragments and moved faster straight-line speeds to and from the forage area than ants without noise exposure. Ants did not recruit fewer foragers under noise conditions as colony activity rates were not impacted. During noise trials leaf size (weight and surface area) was consistently reduced from the first time point through to the final recording point in each replicate. Furthermore, consistent differences in leaf size throughout the replicates suggest the colony did not become habituated to the experimental noise treatment. Experimental exposure to noise had clear effects on the foraging behaviour and resource acquisition of leafcutter ants, which may limit the colony’s fitness over time.

Exposure to noise disturbance can cause stress in animals that manifests itself in a variety of physiological features, such as elevated metabolic rates [31, 32], emission of stress hormones [33, 34] and impacts on movement cohesion and speed [35, 36]. Leafcutter ants in the current study exhibited an increase in straight-line movement speed which could perhaps reflect a stress response to escape a perceived threat. This physical response to move from a threat or disturbance when foraging would generally be anticipated, but it is notable in this case that the resource task is still completed under adverse conditions. This corresponds with research on aquatic invertebrates where resource acquisition persists under elevated noise conditions but with less desirable outcomes, for example, hermit crab (*Pagurus bernhardus)* choosing inferior shells [35], and increased prey handling time in damselfly larvae (*Ischnura elegans)* [37]. The pressure to continue resource tasks in elevated noise conditions is analogous to the starvation versus predation risk trade-off when foraging, which has been well studied in certain vertebrate groups [38], and may explain why inferior resource acquisition continues with noise disturbance in these invertebrate groups.

Increased movement speed on its own seems a beneficial behaviour as leaf fragments are returned to the nest faster, but quicker movement speed under noise treatments resulted in smaller leaf fragments. To achieve faster carrying speeds leafcutter ants reduce the size of transported fragments and this reduces the overall colony leaf transportation rates [39]. Cutting smaller leaf fragments may also be a strategy of reducing the exposure time to noise disturbance, as smaller leaves take less time to cut [25]. Reducing fragment size has also been experimentally demonstrated in favour of increased worker recruitment [25] and increased trails gradients [16]. This flexibility in movement speed and fragment size may be a plastic response enabling the continued leaf transport under adverse or disturbed conditions. It remains uncertain if total energetic cost to the colony would be increased as previous work has demonstrated that 90% of the leaf cutting for two *Atta* species took place inside the nest, and less energy was required to process smaller fragments within the nest [15]. However, any continued disruption to the colony’s energetic balance over time could result in a decrease in food supply, which may lead to negative colony impacts such as reduced population size.

It is likely that noise impacted several sensory modalities in leafcutter ants in our study. The noise produced through vibration speakers (substrate vibrations and airborne pressure waves) are likely to impact conspecific communication via disruption to stridulation signalling. Stridulation is important in several aspects of ant foraging, both in recruitment and defence [20]. Although some debate exists regarding ants’ capacity to perceive airborne sound, including conspecific stridulation [40, 41], ants do have the ability to perceive substrate vibration [20] and in the current study ants appear to have adapted and coordinated their foraging strategy within the first ten minutes of recording. Whether this involved conspecific communication through stridulation and substrate vibration, perhaps during pauses in the intermittent noise, or possibly in the laying of pheromone trails which are used to modulate worker recruitment [42], remains unknow and warrants further investigation. However, it seems unlikely that the ants could communicate using stridulation during the bursts of sound as the volume of the stimuli was substantial and likely to mask any conspecific communication, similar to birds near an aircraft runway [43]. However, the ability to maintain colony activity and rapidly regulate the colony’s movement speed is critical in maintaining leaf transportation rates. Experiments have demonstrated bottlenecking and reduced transport along forage trails, when ants are unequally loaded and moving at different speeds [44].

It is interesting to note that the colony reduced leaf size from the first time point during elevated noise exposure and this reduction remains consistent throughout the time points in each replicate (Fig 3). Furthermore, this consistency in reduced leaf size fragments was recorded over the length of the experiment for noise treatments, whereas controls showed more variation in leaf size fragments (S1 Fig). This suggests that the colony did not habituate to the noise treatments across the length of the experimental period. But also, this implies that individual foragers used external signals or conspecific communication from initial scouts to determine fragment size, rather than gradual information feedback driving gradual behavioural change. Similarly, Norton *et al*. [24] found that leaf load size was determined at the onset of foraging rather than more progressively through the foraging event in their experiments on manipulated leafcutter trail gradients. Norton *et al*. [24] suggest that this behavioural modification was therefore based on an individual’s experience rather than colony-level feedback. The observation that control replicates in the current study elicited a more varied response in leaf metrics suggests that perhaps other non-recorded signals played a part in leaf fragment selection under ambient conditions. For example, while the forage offered remained consistent in source and volume, perhaps natural variation in leaf nutrients, water content or secondary metabolites affected the variation in forage size under non-noise conditions [17, 45].

While this study demonstrates consistent behavioural changes in movement speed and leaf size fragments in response to elevated noise, the study only investigated a single colony. It is likely that colony size, age, composition of worker morphologies, as well as individual and group personality are likely to impact responses to disturbances such as those caused by elevated noise [23, 26, 46]. The morphological composition of colonies can differ and different worker morphologies relate to different leaf harvesting capacity [23]. Furthermore, when Argentine ant (*Linepithema humile*) groups consist of a higher proportion of ‘exploratory’ individuals they display improved speed and accuracy of nest site selection [46]. To demonstrate a wider species consistent response to white noise, or to test how colony personality may impact the range of responses, our study would need to be repeated across multiple colonies.

In conclusion, our study shows the impact noise has on resource acquisition of *A*. *octospinosus*, which corresponds to noise reduced efficiency in other invertebrate taxa [35, 37, 47]. Masking of communication during noise treatments may have impacted resource acquisition. Implications for such impacts over time would likely cause significant detriment to colony fitness. To our knowledge, this is the first study to show the impacts of noise disturbance on the foraging efficiency of social insects and it generates several important questions that need further investigation. Future work should ascertain how different sensory modalities are used to detect distinct noise signals, how noise affects conspecific communication, and ultimately, to what extent does noise impact colony fitness in social invertebrates.

## Supporting information

S1 Fig Mean (±SD) of foraged leaf fragment A) surface area (cm^2^) and B) dry mass (g) by *A*. *octospinosus* under noise and control treatments recorded across the ten experiment replicates.
(PDF)
