# Supplementary material for: Leafcutter ants adjust foraging behaviours when exposed to noise disturbance
Source: PLoS One. 2022 Jun 8;17(6):e0269517. doi: 10.1371/journal.pone.0269517 (PMC9176835; doi:10.1371/journal.pone.0269517)
Supplement: S1 Fig — Mean (±SD) of foraged leaf fragment A) surface area (cm2) and B) dry mass (g) by A. octospinosus under noise and control treatments recorded across the ten experiment replicates. (PDF) [file pone.0269517.s001.pdf]

Supporting information

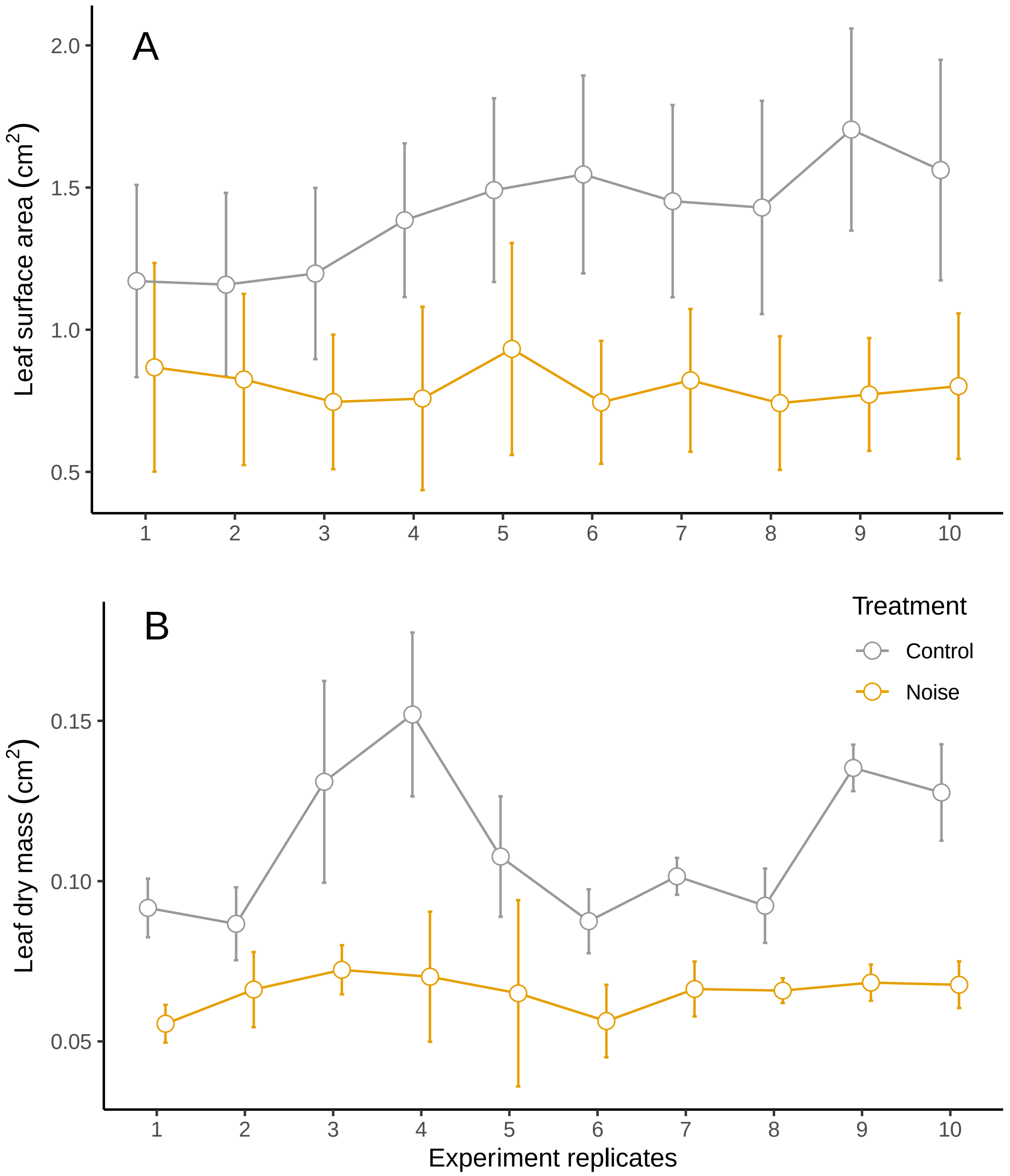

S1 Fig. Mean (±SD) of foraged leaf fragment A) surface area (cm^2) and B) dry mass (g) by *A. octospinosus* under noise and control treatments recorded across the ten experiment replicates.
